# Acute kidney injury associated with febuxostat and allopurinol: a post-marketing study

**DOI:** 10.1186/s13075-019-2011-y

**Published:** 2019-11-08

**Authors:** Amayelle Rey, Benjamin Batteux, Solène M. Laville, Justine Marienne, Kamel Masmoudi, Valérie Gras-Champel, Sophie Liabeuf

**Affiliations:** 1 Regional Pharmacovigilance Centre, Division of Clinical Pharmacology, Amiens University Hospital, Amiens, France; 2 MP3CV Laboratory, EA7517, University of Picardie Jules Verne, F-80000 Amiens, France; 3 CESP Centre for Research in Epidemiology and Population Health, Université Paris-Saclay, Université Paris Sud, UVSQ, UMRS 1018, F-94807 Villejuif, France; 4 Clinical Pharmacology Division, Amiens University Medical Center, Avenue René Laennec, F-80000 Amiens, France

**Keywords:** Acute renal failure, Urate-lowering therapies, Pharmacovigilance, Post-marketing study

## Abstract

**Background:**

For patients with recurrent flares of gout, tophi, urate crystal arthropathy, and renal stones, urate-lowering therapies (ULTs, including allopurinol and febuxostat) are the first-line treatment. Due to the widespread use of these ULTs (especially in patients with impaired renal function), assessment of the associated renal risk is essential. Accordingly, we performed a disproportionality analysis of reported cases of acute renal failure (ARF) associated with allopurinol and febuxostat.

**Methods:**

We carried out a case/non-case study of the World Health Organization’s VigiBase® pharmacovigilance database between January 1, 2008, and December 31, 2018. The frequency of reports of ARF as a standardized Medical Dictionary for Regulatory Activities query for allopurinol and febuxostat was compared with that of all other reports for the two drugs and quoted as the reporting odds ratio (ROR) [95% confidence interval (CI)]. The results’ stability was assessed in a series of sensitivity analyses (notably after the exclusion of putative competing drugs).

**Results:**

Among 3509 “suspected drug” notifications for febuxostat and 18,730 for allopurinol, we identified respectively 317 and 1008 cases of ARF. Acute renal failure was reported significantly more frequently for febuxostat and allopurinol than for other drugs (ROR [95%CI] 5.67 [5.05–6.36] and 3.25 [3.05–3.47], respectively). For both drugs, the ROR was higher in women than in men, respectively 11.60 [9.74–13.82] vs. 3.14 [2.69–3.67] for febuxostat and 4.45 [4.04–4.91] vs. 2.29 [2.11–2.50] for allopurinol. The sensitivity analyses confirmed the disproportionality for these two ULTs.

**Conclusions:**

Acute renal failure was reported respectively 5.7 and 3.3 times more frequently for febuxostat and for allopurinol than for other drugs. Due to the potential consequences of ARF, physicians should take account of this disproportionality signal when prescribing the ULTs febuxostat and allopurinol.

## Background

Gout is the most common type of inflammatory arthritis. Although the reported prevalence of gout varies greatly from one country to another (~ 0.1 to ~ 10%), a value above 1% is found in North America, Europe, and most developed countries [1]. The prevalence of gout increases with age, and the male to female ratio is generally between 3:1 and 4:1. Gout is associated with a high frequency of comorbidities (especially renal and cardiovascular diseases), which worsens patients’ prognosis and complicates their treatment [2]. In view of the elevated prevalence, misdiagnosis, and suboptimal treatment [3], several learned societies (such as the European League Against Rheumatism [4] and the American College of Rheumatology [5]) have revised their guidelines on the management of chronic gout. For patients with recurrent flares of gout, tophi, urate crystal arthropathy, and kidney stones, urate-lowering therapies (ULTs) are still the recommended first-line treatment.

The oldest and most frequently prescribed ULT is allopurinol, which was first marketed in 1966. The second most frequently prescribed ULT is febuxostat, which has been authorized in France since 2008. In both cases, the mechanism of action is based on xanthine oxidase (XO) inhibition [6]. The purine analog allopurinol is a substrate for XO. The main metabolite is oxypurinol—an XO inhibitor and predominantly responsible for allopurinol’s therapeutic action. Febuxostat is a potent, selective non-purine inhibitor of XO and does not influence the activity of other enzymes involved in purine or pyrimidine pathways [6].

Choosing between these two drugs often depends on the patient’s renal status because allopurinol is mostly excreted by kidneys [7]. Impaired renal function may cause the retention of allopurinol and its metabolites, which consequently prolongs their plasma half-lives and increases the risk of serious adverse drug reaction adverse events, such as allopurinol-induced Stevens-Johnson syndrome and toxic epidermal necrolysis [8, 9]. In contrast, febuxostat is mostly excreted by the liver, and so, dose level adjustment is not required in patients with mild-to-moderate renal impairment [10]. However, febuxostat’s efficacy and safety have not been extensively evaluated in patients with severe renal impairment (i.e., a creatinine clearance rate below 30 mL/min) [11–14]; the published studies were performed in small numbers of patients (e.g., only 36 patients with late-stage CKD) and over short periods [15].

Drugs constitute one of the leading causes of acute kidney injury (AKI) [16]. The prevalence of drug-induced AKI has increased in recent years [17, 18]—especially in intensive care units. According to the meta-analysis performed by Cartin-Ceba et al., each additional nephrotoxic drug increases the risk of developing AKI by 53% [19]. In fact, many known nephrotoxic drugs (such as non-steroidal anti-inflammatory drugs (NSAIDs), diuretics, and angiotensin-converting enzyme inhibitors (ACEIs) [17, 20] are prescribed for the treatment of acute gout flare or for comorbidities [4]. Although ULTs have sometimes been presented as renoprotective by targeting hyperuricemia [21], renal risks associated with allopurinol or febuxostat have rarely been evaluated, especially in post-marketing studies.

The primary objective of the present study was to investigate the putative renal risk associated with two ULTs (febuxostat and allopurinol). To this end, we searched for a disproportionality signal in a large international pharmacovigilance database.

## Materials and methods

### Data source

Individual case safety reports (ICSRs) from VigiBase® (the World Health Organization (WHO)’s global database of suspected adverse drug reactions (ADRs)) were analyzed in a case/non-case disproportionality study. VigiBase® was established in 1968 and has been managed by the Uppsala Monitoring Centre [22] since 1978. It is the largest data resource of its kind in the world and contains over 20 million ICSRs on suspected ADRs submitted by more than 150 countries participating in the WHO’s Program for International Drug Monitoring. The anonymized ICSRs are variously submitted by health professionals, patients, and pharmaceutical companies in the member countries. VigiBase® is continuously updated with the incoming ICSRs.

Each ICSR includes anonymous administrative data (the country, and the reporter’s qualification), patient information (age and gender), drug information (the international non-proprietary name or trade name, Anatomical Therapeutic Chemical (ATC) classification, indication, start date, withdrawal date, dosage, and administration route), and information on the suspected ADR (coded according to the Medical Dictionary for Regulatory Activities (MedDRA)) [23]. There are five levels to the MedDRA hierarchy, ranging from very general to very specific. MedDRA also includes standardized MedDRA queries (SMQs), which are collections of MedDRA terms consistent with a description of a clinical syndrome associated with ADR and drug exposure. As such, SMQs are useful for wide-ranging searches. If a drug is considered to be at least probably responsible for the adverse event, it is defined as “suspect” or “interacting” in the ICSR. If not, it is defined as “concomitant.” Probability scales are based on a variety of chronologic, semiologic, and/or bibliographic criteria [24]. Full information on ICSRs is given on the Uppsala Monitoring Centre’s website [25].

An ADR is categorized as “serious” if it results (at any dose) in an untoward medical occurrence, such as (from the least serious to the worst serious) initial or prolonged hospitalization, persistent or significant disability/incapacity, a life-threatening event, or death.

### Selection of cases and non-cases

We selected ADRs recorded in VigiBase® between January 1, 2008, and December 31, 2018, and excluded ADRs occurring in patients under the age of 18 and in those whose age or gender was not specified.

For cases, the primary analysis included all VigiBase® ICSRs detected with an SMQ of “acute renal failure” (ARF) and for which febuxostat (ATC code: M04AA03) or allopurinol (ATC code: M04AA01) was the “suspected” drug. This broad SMQ was chosen so as not to exclude cases of ARF coded with another MedDRA term (Additional file 1: Table S1). The SMQ notably includes the MedDRA term “acute kidney injury,” which is the new consensus term for ARF [26]. It focuses on the acute (i.e., sudden) potentially reversible failure of kidney function, and MedDRA terms for prolonged renal failure were excluded. In addition, we previously demonstrated that this SMQ identifies correctly cases of AKI [16].

### Statistical analyses

Descriptive statistics were used to summarize the baseline characteristics of the cases for febuxostat and allopurinol.

We performed a primary case/non-case analysis in which the disproportionality between ARF and all other ADRs was expressed as the reporting odds ratio (ROR) [95% confidence interval (CI)] for febuxostat and allopurinol vs. all other drugs (Additional file 1: Table S2). If the ROR and the lower boundary of the 95%CI are greater than 1, the ADR of interest is reported more frequently with the drug of interest than with all other drugs. Moreover, it has been suggested that an ROR greater than 4.0 corresponds to a “large” effect size [27]. In the present case/non-case study, non-cases corresponded to all cases of ARF due to another drug than febuxostat or allopurinol spontaneously reported to VigiBase® during the study period.

Secondary analyses were performed by calculating the ROR by region, age category, sex, and gout indication. To manage a possible bias due to a Weber effect (i.e., a peak in ADR reporting in the two first years after approval) for febuxostat (authorized in 2008), we also performed a ROR by year of notification from 2011 onwards [28, 29]. We performed various sensitivity analyses. Firstly, we repeated the primary analysis for ICSRs submitted by healthcare professionals only. This analysis was used to control for potential misclassification errors in ICSRs that had not been medically confirmed. To minimize any competition bias and underestimation [30–32], we excluded drugs known to induce ARF (i.e., NSAIDs, diuretics, ACEIs, and angiotensin II receptor antagonists) from all reported ARF cases and from all ICSRs [33]. In order to reduce indication bias [30, 31, 34] (i.e., the possibility that febuxostat was used preferentially in CKD patients), we compared febuxostat directly with other drugs in the same class (i.e., other XO inhibitors). To assess the stability of our results, analyses were performed by selecting ibuprofen as a positive control (since this drug is known to be associated with ARF) and alprazolam as a negative control [31].

## Results

**Fig. 1:**
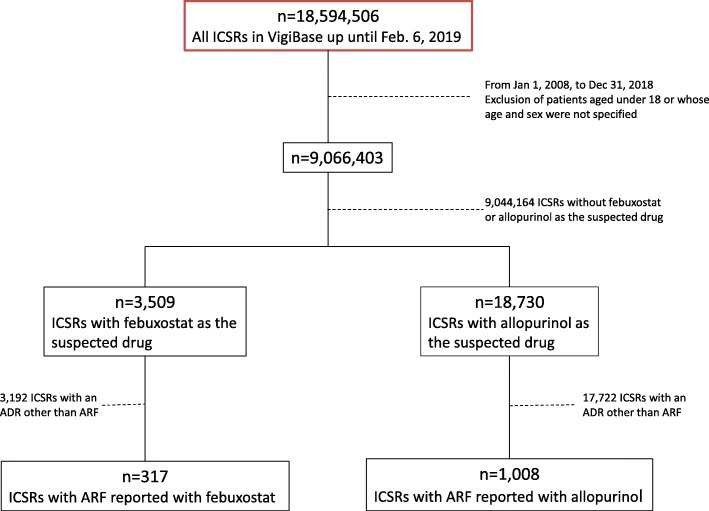
Study flowchart

Of the 9,066,403 ICSRs in adult patients of known age and gender reported to VigiBase® between January 1, 2008, and December 31, 2018, 3509 concerned febuxostat and 18,730 concerned allopurinol. Respectively 317 and 1008 ICSRs for febuxostat and allopurinol matched the SMQ for ARF (Fig. 1).

The baseline characteristics of the study population specifically presenting ARF with the two drugs of interest are summarized in Table 1.

**Table 1:** Baseline characteristics of study cases with febuxostat and allopurinol

Characteristics	Acute renal failure with febuxostat as the suspected drug (*n* = 317)	Acute renal failure with allopurinol as the suspected drug (*n* = 1008)
Age, years, mean (SD)	68.0 (15.0)	67.0 (14.5)
Age, *n* (%)		
19–44	27 (8.5)	79 (7.8)
45–64	83 (26.2)	306 (30.4)
65–74	81 (25.6)	270 (26.8)
≥ 75	126 (39.7)	353 (35.0)
Sex, *n* (%)		
Male	172 (54.3)	577 (57.2)
Female	145 (45.7)	431 (42.8)
Reporter qualification, *n* (%)		
Healthcare professional	281 (88.6)	863 (85.6)
Not a healthcare professional	17 (5.4)	41 (4.1)
Unknown	19 (6.0)	104 (10.3)
The only suspected drug, *n* (%)	184 (58.0)	447 (44.3)
Continent, *n* (%)		
North America	115 (36.3)	233 (23.1)
Europe	115 (36.3)	489 (48.5)
Asia	82 (25.9)	237 (23.5)
Other regions*	5 (1.6)	49 (4.9)
Seriousness of ARF		
Death	22 (6.9)	97 (9.6)
Life-threatening event	32 (10.1)	111 (11.0)
Disability/incapacity	18 (5.7)	13 (1.3)
Hospitalization (caused or prolonged)	115 (36.3)	482 (47.8)
Others	70 (22.1)	111 (11.0)
Unknown	60 (18.9)	194 (19.2)

*Included Oceania and Africa

Most of the analyzed cases of ARF due to febuxostat or allopurinol had been notified by healthcare professionals (88.6% and 85.6%, respectively). The cases predominantly occurred in men (54.3% for febuxostat and 57.2% for allopurinol). The mean ± standard deviation age was 68.0 ± 15.0 for cases of ARF linked to febuxostat and 67.0 ± 14.5 for those linked to allopurinol. With regard to the outcome, the mortality rate was 6.9% and 9.6% for cases reported for febuxostat and allopurinol, respectively. Most of the notified cases of ARF came from Europe and North America for febuxostat and from Europe for allopurinol.

Febuxostat was the sole “suspected drug” in 58.0% of the ICSRs with ARF in febuxostat-treated patients. The corresponding value for allopurinol was 44.3%. In the other ICSRs, the other “suspected drugs” in febuxostat-treated patients were anti-anemics or antihemorrhagics (11.4%), diuretics (10.3%), lipid-lowering agents (6.9%), immunosuppressive therapies (6.6%), calcium channel blockers (5.9%), antigout drugs (5.5%), and NSAIDs (3.0%) (Table 2). The other “suspected drugs” in allopurinol-treated patients were diuretics (11.3%), antibiotics (10.7%), immunosuppressive therapies (7.5%), ACEIs (5.1%), antigout drugs (4.6%), sartans (4.6%), and NSAIDs (4.0%) (Table 2). A gout indication was reported for 47.0% of febuxostat ICSRs and 28.8% for allopurinol ICSRs. However, in the other indications, missing data and non-specified hyperuricemia were respectively reported in 20.8% and 30.0% of febuxostat ICSRs and 45.3% and 18.0% of allopurinol ICSRs.

**Table 2:** Main suspected drug classes and drugs associated with febuxostat/allopurinol in reported cases of ARF

	Number	Percent
Febuxostat		
Anti-anemics/antihemorrhagics	64	11.4
Darbepoetin alfa	43	7.7
Diuretics	58	10.3
Furosemide	25	4.4
Hydrochlorothiazide	8	1.4
Lipid-lowering agents	39	6.9
Rosuvastatin	9	1.6
Simvastatin	7	1.2
Immunosuppressive therapies	37	6.6
Tocilizumab	15	2.7
Calcium channel blockers	33	5.9
Amlodipine	16	2.8
Nifedipine	7	1.2
Antigout drugs	31	5.5
Colchicine	15	2.7
Allopurinol	14	2.5
NSAIDs	17	3.0
Diclofenac	5	0.9
Ketoprofen	3	0.5
Allopurinol		
Diuretics	247	11.3
Furosemide	107	4.9
Hydrochlorothiazide	52	2.4
Spironolactone	37	1.7
Antibiotics	234	10.7
Amoxicillin	27	1.2
Sulfamethoxazole/trimethoprim	20	0.9
Immunosuppressive therapies	163	7.5
Tocilizumab	15	0.7
Cyclosporine	11	0.5
Lenalidomide	11	0.5
ACEIs	112	5.1
Perindopril	31	1.4
Ramipril	26	1.2
Antigout drugs	101	4.6
Colchicine	75	3.4
Febuxostat	14	0.6
Sartans	100	4.6
Valsartan	28	1.3
Irbesartan	19	0.9
NSAIDs	87	4.0
Ibuprofen	21	1.0
Diclofenac	15	0.7

**Fig. 2:**
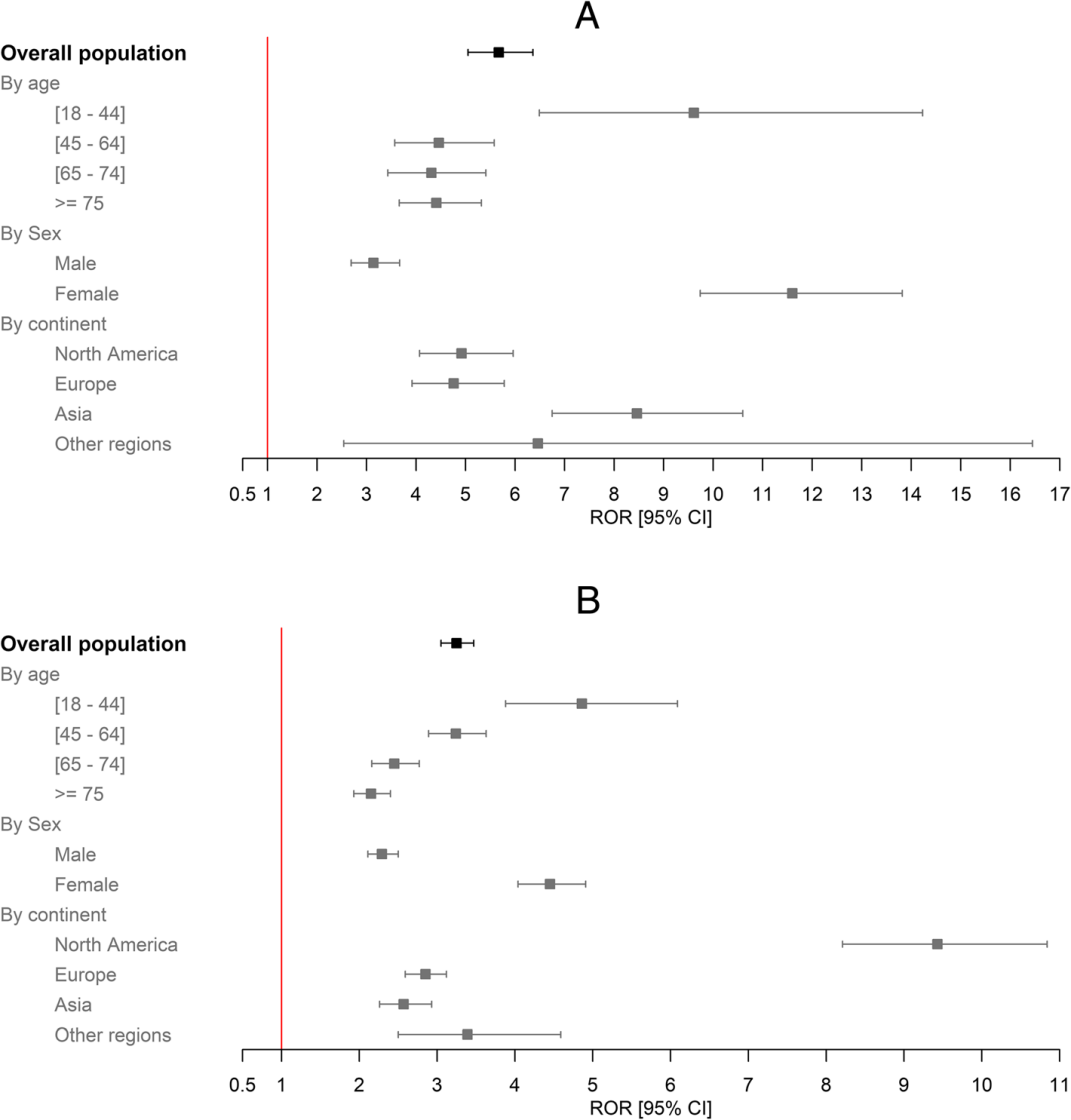
RORs for ARF with febuxostat (**a**) and allopurinol (**b**) depending on the evaluation criteria

The RORs [95%CI] for ARF due to febuxostat and allopurinol were respectively 5.67 [5.05–6.36] and 3.25 [3.05–3.47] (Fig. 2a, b).

The secondary analyses were in line with the primary analysis and highlighted disproportionality (Fig. 2a, b). The highest RORs by region were found in Asia (8.46 [6.75–10.60] for febuxostat) and North America (9.43 [8.21–10.84]) for allopurinol). For both drugs, the ROR was higher in women than in men, respectively 11.60 [9.74–13.82] vs. 3.14 [2.69–3.67] for febuxostat and 4.45 [4.04–4.91] vs. 2.29 [2.11–2.50] for allopurinol. When considering the various age classes, the highest ROR was in the 18–44 class for both drugs. When we limited the analysis to the indication of gout, the RORs for febuxostat and allopurinol were respectively 2.53 [2.14–2.98] and 0.90 [0.80–1.01]. The per-year RORs for both drugs were significant and stable (Additional file1: Table S3), with the exception of a peak in the ROR for febuxostat between 2011 and 2013 (e.g., 14.81 [9.82–22.34] in 2011).

The sensitivity analyses of ICSRs reported by health professionals only gave the same results as the primary analysis. When febuxostat or allopurinol was the only suspected drug, the ROR was still significant (3.15 [2.72–3.66] and 1.39 [1.27–1.53], respectively). The ROR was even greater when we excluded ICSRs involving the drugs mostly associated with ARF; the values were 6.34 [5.65–7.11] for febuxostat and 3.64 [3.42–3.88] for allopurinol (Table 3). When we set the reference group to “other XO inhibitors,” the ROR for febuxostat was 1.74 [1.53–1.99].

**Table 3:** RORs for risk of ARF with febuxostat/allopurinol, after excluding drugs frequently associated with ARF

Exposure	ARF	Non-ARF	ROR	95%CI
Febuxostat	317	3192	*6.34*	*5.65–7.11*
Allopurinol	1008	17,722	*3.64*	*3.42–3.88*

The ROR was 26.93 [25.49–28.44] for the positive control (ibuprofen) and 0.40 [0.34–0.47] for the negative control (alprazolam).

## Discussion

In the present study, we detected a disproportionality signal for ARF and the ULTs febuxostat and allopurinol. The presence of this signal was confirmed in a variety of sensitivity analyses (i.e., when febuxostat and allopurinol were the only suspected drugs, when we used other XO inhibitors as the comparator or when known nephrotoxic drugs were excluded).

The purpose of studying the disproportionality of spontaneous ICSRs is to generate pharmacovigilance alerts concerning unknown or underestimated ADRs. Until the present case/non-case study, the risk of AKI associated with allopurinol and febuxostat had been underestimated. In fact, renal and urinary tract disorders were reported (albeit not in detail) during an extended phase III febuxostat clinical trial [12]. However, AKI has not been mentioned for other phase III trials [11, 13, 14]. Moreover, to the best of our knowledge, no phase IV studies have addressed this topic for the two studied ULTs.

The main known risk factors for AKI are underlying kidney disease, diabetes, cardiovascular disease, and acute situations such as dehydration [2]. Adverse drug reactions are also a common cause of AKI; many compounds can induce or aggravate the condition [17]. The present pharmacovigilance alert is of particular interest for populations more prone to develop AKI, such as older adults or patients with chronic kidney disease (CKD). Several studies have already highlighted the relationship between CKD and acute kidney injury (AKI) [35, 36]. On one hand, AKI may contribute to the development and progression of CKD. On the other hand, CKD is known to predispose or sensitize patients to AKI and to slow down the kidneys’ ability to recover [37]. We now know that CKD can induce hyperuricemia, since uric acid (the main biomarker of gout) is excreted primarily by the kidney [38]. Given than (i) ULTs are frequently prescribed to CKD patients prone to hyperuricemia [39] and (ii) the risk of AKI is elevated in CKD patients, our present findings are of value for physicians who manage patients with impaired renal function. The prescription of ULTs (especially allopurinol) to patients with CKD is subject to much debate. Although some researchers consider that dose adjustment is a valid means of reducing the incidence of allopurinol-induced ADRs (such as dermatologic ADRs), other researchers disagree [40–42]. Our present finding of disproportionality for ARF and allopurinol should further prompt physicians to review their prescription of this drug in patients prone to AKI (such as patients with CKD). Furthermore, two recent reports have highlighted febuxostat’s renoprotective effect [43, 44], and asymptomatic hyperuricemia has been presented as a potential therapeutic target in patients with CKD [21]. Even though the VigiBase® data do not enable the specific identification of patients with CKD, ARF was more reported 5.67 times more frequently with febuxostat than with other drugs; this signal persisted in sensitivity analysis limited to the indication of gout. This signal needs to be taken into account before extending the use of febuxostat to asymptomatic hyperuricemia in patients with CKD. Pharmacological and randomized studies are necessary to understand the physiopathology and causal nature of febuxostat- and allopurinol-induced AKI. Physicians might wonder whether this ADR is likely to occur in their patients. In fact, the small number of corresponding ICSRs in international databases suggests that this reaction is rare. However, we believe that it is important to inform allopurinol and febuxostat prescribers about this potential ADR—especially when they are treating at-risk patients (i.e., women and patients with CKD).

A significant ROR was found for each subgroup studied in our secondary analyses. Women had a higher ROR than men—especially for febuxostat. A number of recent literature results are controversial; although most clinical studies predominantly include male patients [45–47], recent findings indicate that specific physiologic features in women might impact the response to injury and/or treatment [45]. Depending on the situation, women might be less protected [47]. Our results suggest that caution is required when considering the prescription of febuxostat and allopurinol in women at risk of AKI. When comparing the two drugs in different regions of the world, the highest ROR was noted in North America (9.43 [8.2–10.8]) for allopurinol and in Asia (8.5 [6.8–10.6]) for febuxostat. These differences might be due to inter-country differences in notification methods and/or ethnic differences in susceptibility to specific ADRs [48, 49]. It is noteworthy that the magnitude of the ROR was relatively constant over time, although higher values were noted from 2011 to 2013 for febuxostat; this might have been due to a Weber effect [28, 29].

Patients with gout are often treated with drugs known to induce AKI, such as diuretics, ACEIs, angiotensin II receptor antagonists, and NSAIDs. To assess the stability of our results, we performed a sensitivity analysis in which these drugs were excluded. The RORs were still significant for both drugs and were even higher than in the primary analysis.

Our study suffers from the inherent limitations of all pharmacovigilance studies [30, 31] and case-control designs. We did not have any data on the number of patients exposed to a particular drug, which prevents one from calculating the absolute frequency of ARF on the basis of ICSRs (notably because of underreporting [50]). Moreover, the data collection differed from one country to another for legislative and regulatory reasons. However, widespread underreporting would not affect the results of this kind of study [30]. Furthermore, it is known that underreporting is similar for drugs within a given class [51], and so, a comparison of allopurinol and febuxostat is possible. Also, pharmacovigilance databases are designed to detect signals, rather than to exhaustively record all ADRs [30, 31]. We did not have any information on over-the-counter medication use. This lack of information might have led to an underestimation of the use of associated drugs known to induce AKI (such as NSAIDs). Certain data on medications are not exhaustively recorded in VigiBase [52]. However, this information bias was anticipated and limited by analyzing only the cases in which sex and age were known. The proportion of missing data was high for some parameters (the indication, in particular); this limitation—inducing a lack of power—might explain why the ROR was no longer significant with allopurinol when the analysis was limited to the indication of gout. Lastly, VigiBase® does not contain comprehensive information on the patient’s medical history, which prevented us from analyzing other risk factors possibly associated with AKI. Likewise, VigiBase® does not contain detailed information on the patient’s clinical status, such as the seriousness or etiology of previous AKI or CKD.

In contrast, our study has several important strengths linked to its case/non-case design [30, 31]. Firstly, we studied the world’s largest pharmacovigilance database. Second, more than 150 countries participate in the WHO Program for International Drug Monitoring, accounting for over 90% of the world’s population. Hence, the data were exhaustive and reflected “real-life” medication use. Thirdly, the case/non-case study is a validated method of investigating disproportionality between reports and drugs. It has already been shown that this kind of study can detect signals for rare ADRs [30, 31]. We choose to calculate ROR as a measure of disproportionality; a study of disproportionality indices in the FDA reporting database concluded that the ROR gives the best results [53]. Furthermore, we checked the consistency of our results using positive and negative controls. We also managed competition bias by excluding ICSRs that involved drugs frequently associated with AKIs because they could distort the signal and lead to false-positive results. For both febuxostat and allopurinol, the signal in the latter analysis was even stronger than in the primary analysis, which reinforces the putative involvement of these drugs in the occurrence of AKI.

## Conclusion

We reported that ARF is respectively 5.7 and 3.3 more frequently reported with febuxostat and allopurinol than with other drugs. Due to the potential consequences of AKI in terms of mortality and renal impairment [36, 54], physicians should take the present signal into account when prescribing febuxostat or allopurinol. Pharmacovigilance databases are very valuable tools for post-marketing surveillance and can be also used to monitor treatment effectiveness in phase IV clinical studies [55]. Our present results pave the way for randomized phase IV trials designed to assess the likely causal relationship between AKI and febuxostat or allopurinol.

## Supplementary information

**Additional file 1: Table S1.** MedDRA terms included in the standardized MedDRA query “acute renal failure”. **Table S2.** Calculation of the ROR. **Table S3.** Reporting odds ratios for the risk of ARF induced by febuxostat and by allopurinol in the overall study population, by year.

## Data Availability

The datasets used and/or analyzed during the current study are available from the corresponding author on reasonable request, subject to obtaining the agreement of Uppsala Monitoring Centre.

## Abbreviations

ACEI: Angiotensin-converting enzyme inhibitors
ADRs: Adverse drug reactions
AKI: Acute kidney injury
ANSM: Agence Nationale de Sécurité du Médicament et des produits de santé (France)
ARF: Acute renal failure
ATC: Anatomical Therapeutic Chemical
CKD: Chronic kidney disease
CI: Confidence interval
ICSRs: Individual case safety reports
MedDRA: Medical Dictionary for Regulatory Activities
NSAIDs: Non-steroidal anti-inflammatory drugs
ROR: Reporting odds ratio
SMQ: Standardized MedDRA queries
ULT: Urate-lowering therapies
WHO: World Health Organization
XO: Xanthine oxydase
