# Supplementary material for: Acute kidney injury associated with febuxostat and allopurinol: a post-marketing study
Source: Arthritis Res Ther. 2019 Nov 8;21:229. doi: 10.1186/s13075-019-2011-y (PMC6842268; doi:10.1186/s13075-019-2011-y)
Supplement: Supplementary file 1 — Additional file 1: Table S1. MedDRA terms included in the standardized MedDRA query “acute renal failure”. Table S2. Calculation of the ROR. Table S3. Reporting odds ratios for the risk of ARF induced by febuxostat and by allopurinol in the overall study population, by year. [file 13075_2019_2011_MOESM1_ESM.docx]

**Additional file 1: Table S1: MedDRA terms included in the standardized MedDRA query “acute renal failure”**

| **MedDRA terms** | |
| --- | --- |
| Acute kidney injury | Hypercreatininaemia |
| Acute phosphate nephropathy | Hyponatriuria |
| Albuminuria | Intradialytic parenteral nutrition |
| Anuria | Kidney injury molecule-1 |
| Azotaemia | Neonatal anuria |
| Blood creatinine abnormal | Nephritis |
| Blood creatinine increased | Nephropathy toxic |
| Blood urea abnormal | Oedema due to renal disease |
| Blood urea increased | Oliguria |
| Blood urea nitrogen/creatinine ratio increased | Peritoneal dialysis |
| Continuous haemodiafiltration | Prerenal failure |
| Creatinine renal clearance abnormal | Protein urine present |
| Creatinine renal clearance decreased | Proteinuria |
| Creatinine urine abnormal | Renal failure |
| Creatinine urine decreased | Renal failure neonatal |
| Crystal nephropathy | Renal function test abnormal |
| Dialysis | Renal impairment |
| Foetal renal impairment (PT narrow) | Renal impairment neonatal |
| Fractional excretion of sodium | Renal transplant |
| Glomerular filtration rate abnormal | Renal tubular disorder |
| Glomerular filtration rate decreased | Renal tubular dysfunction |
| Haemodialysis | Renal tubular injury |
| Haemofiltration | Renal tubular necrosis |
| Tubulointerstitial nephritis | Urea renal clearance decreased |
| Urine output decreased |  |

**Additional file 1: Table S2: Calculation of the ROR.**

|  | ADRs with ARF | All other ADRs | Total |
| --- | --- | --- | --- |
| Drug of interest | a | b | a + b |
| Other drugs | c | d | c + d |

*a: the number of exposed cases (ARF with the drug of interest, i.e. febuxostat or allopurinol); b: the number of exposed non-cases (all ADRs other than ARF reported for the drug of interest); c: the number of non-exposed cases (ARF with all other drugs); d: the number of unexposed non-cases (all ADRs other than ARF, with other drugs). The total number of ADRs reported between January 1, and December 31, 2018, was a + b + c + d. The ROR is defined as (*a*/*c*)/(*b*/*d*) =* ad*/*bc*.*

**Additional file 1: Table S3: Reporting odds ratios for the risk of ARF induced by febuxostat and by allopurinol in the overall study population, by year.**

| **Exposure** | **ARF** | **Non-ARF** | **ROR** | **95%CI** |
| --- | --- | --- | --- | --- |
| **Febuxostat** |  |  |  |  |
| By year |  |  |  |  |
| 2011 | 30 | 95 | 14.81 | 9.82-22.34 |
| 2012 | 22 | 112 | 10.72 | 6.79-16.95 |
| 2013 | 33 | 191 | 8.42 | 5.82-12.19 |
| 2014 | 39 | 371 | 6.14 | 4.41-8.54 |
| 2015 | 94 | 1,147 | 5.42 | 4.39-6.70 |
| 2016 | 38 | 407 | 6.14 | 4.40-8.56 |
| 2017 | 34 | 427 | 5.02 | 3.34-7.12 |
| 2018 | 19 | 377 | 3.46 | 2.18-5.49 |
| **Allopurinol** |  |  |  |  |
| By year |  |  |  |  |
| 2008 | 72 | 765 | 3.47 | 2.72-4.42 |
| 2009 | 46 | 1,034 | 1.73 | 1.29-2.33 |
| 2010 | 66 | 787 | 3.64 | 2.83-4.69 |
| 2011 | 114 | 1958 | 2.74 | 2.21-3.31 |
| 2012 | 16 | 1,150 | 3.05 | 2.37-3.92 |
| 2013 | 82 | 1,190 | 3.37 | 2.69-4.22 |
| 2014 | 164 | 2,544 | 3.78 | 3.23-4.43 |
| 2015 | 117 | 2,029 | 3.82 | 3.17-4.60 |
| 2016 | 95 | 2,841 | 2.20 | 1.79-2.70 |
| 2017 | 102 | 1,823 | 3.54 | 2.89-4.32 |
| 2018 | 86 | 1,601 | 3.70 | 2.97-4.60 |
